# Quantification of cardiac pumping mechanics in TAVI patients: A pilot study utilizing minimally invasive method for pressure‐volume analysis

**DOI:** 10.14814/phy2.15799

**Published:** 2023-09-09

**Authors:** Tsung‐Yu Ko, Chia‐Chuan Chuang, Mao‐Shin Lin, Yi‐Chang Chen, Ying‐Hsien Chen, Ching‐Chang Huang, Chih‐Fan Yeh, Ming‐Jiuh Wang, Kuo‐Chu Chang, Yi‐Lwun Ho, Hsien‐Li Kao

**Affiliations:** ^1^ Division of Cardiology, Department of Internal Medicine and Cardiovascular Center National Taiwan University Hospital Taipei Taiwan; ^2^ Graduate Institute of Clinical Medicine National Taiwan University Taipei Taiwan; ^3^ Department of Radiology National Taiwan University Hospital Taipei Taiwan; ^4^ Department of Anesthesiology National Taiwan University Hospital Taipei Taiwan; ^5^ Department of Physiology, College of Medicine National Taiwan University Taipei Taiwan

**Keywords:** aortic stenosis, pressure‐volume analysis, transcatheter aortic valve implantation, ventriculo‐arterial coupling

## Abstract

The ventriculo‐arterial coupling (VAC) and left ventricle (LV) mechanics are crucial and play an important role in the pathophysiology of aortic stenosis (AS). The pressure‐volume (PV) analysis is a powerful tool to study VAC and LV mechanics. We proposed a novel minimally‐invasive method for PV analysis in patients with severe AS receiving transcatheter aortic valve implantation (TAVI). Patients with severe AS were prospectively enrolled in a single center. LV pressure and cardiac output were recorded before and after TAVI. We constructed the PV loop for analysis by analyzing LV pressure and the assumed flow. 26 patients were included for final analysis. The effective arterial elastance (Ea) decreased after TAVI (3.7 ± 1.3 vs. 2.9 ± 1.1 mmHg/mL, *p* < 0.0001). The LV end‐systolic elastance (Ees) did not change immediately after TAVI (2.4 ± 1.3 vs. 2.6 ± 1.1 mmHg/mL, *p* = 0.3670). The Ea/Ees improved after TAVI (1.8 ± 0.8 vs. 1.2 ± 0.4, *p* < 0.0001), demonstrating an immediate improvement of VAC. The stroke work (SW) did not change (7669.6 ± 1913.8 vs. 7626.2 ± 2546.9, *p* = 0.9330), but the pressure‐volume area (PVA) decreased (14469.0 ± 4974.1 vs. 12177.4 ± 4499.9, *p* = 0.0374) after TAVI. The SW/PVA increased after TAVI (0.55 ± 0.12 vs. 0.63 ± 0.08, *p* < 0.0001) representing an improvement of LV efficiency. We proposed a novel minimally invasive method for PV analysis in patients with severe AS receiving TAVI. The VAC and LV efficiency improved immediately after TAVI.

## Graphical abstract

Reconstructed pressure volume loop by LV pressure and cardiac output.

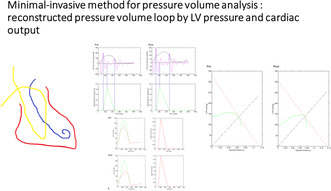

## 1 INTRODUCTION

Aortic stenosis (AS) is an important valvular heart disease and has grave prognosis when it becomes symptomatic (Turina et al., 1987). The parameters of echocardiographic are used to determine the severity of AS (Rosenhek et al., 2000). These parameters including mean pressure gradient, estimated aortic valve area and left ventricular ejection fractions are also important predictors for clinical outcomes and have been validated in many studies (Otto et al., 1997; Rosenhek et al., 2004). However, there is a lot of overlap between symptomatic and asymptomatic patients even with exhaustive echocardiography. The conventional measurements for AS are too simplistic and does not take the interaction between left ventricle (LV), stenotic valve and the arterial system for consideration.

The fundamental pathophysiology of AS is LV dysfunction caused by excessive afterload (Hachicha et al., 2009). Various parameters have been proposed to describe the load condition of LV including valvulo‐arterial impedance (Zva) and systemic arterial compliance (SAC) (Briand et al., 2005; Lancellotti et al., 2010). Zva reflects the sum of valvular and arterial factors that against LV, while SAC reflects the load caused by arterial systems alone. There is little information with respect to the interaction of LV performance and load condition in AS.

Pressure‐Volume (PV) analysis can be used to study the performance of LV and the ventriculo‐arterial coupling (VAC) which represents the interaction of LV and afterload (Suga & Sagawa, 1974). The LV and arterial system are considered as elastic chambers with an intrinsic elastance. End‐systolic elastance (Ees) represents the LV Ees, and effective arterial elastance (Ea) represents the effective arterial elastance (Bastos et al., 2020). Traditional PV analysis needs simultaneous measurement of LV pressure and flow over a range of load‐condition, its clinical utilization is precluded due to the invasiveness (Burkhoff, 2013). We proposed a novel minimally invasive method to study the VAC and LV mechanics in patients with severe AS receiving transcatheter aortic valve implantation (TAVI) (Wang et al., 2019).

## 2 METHODS

### 2.1 Study population

26 patients with symptomatic severe AS undergoing TAVI were enrolled. The clinical and echocardiographic data were prospectively collected. The protocol of this study was approved by the local institutional review board, and all subjects had signed the informed consent (approval number: 202004118RIND).

### 2.2 TAVI procedure and hemodynamic measurement

TAVI was performed by transfemoral approach under conscious sedation or general anesthesia accordingly. Right heart pressure and cardiac output were recorded by Swan‐Ganz catheter at the beginning and the end of TAVI, simultaneously LV and aortic‐root pressure recording were also measured by a fluid‐filled system as well. The signal of pressure and surface electrocardiogram were recorded in a hemodynamic system (Mac‐Lab, GE healthcare), and the hemodynamic data was subsequently sampled and analyzed by MATLAB (MathWorks Inc.).

### 2.3 Construction of the PV loop for analysis

**FIGURE 1:**
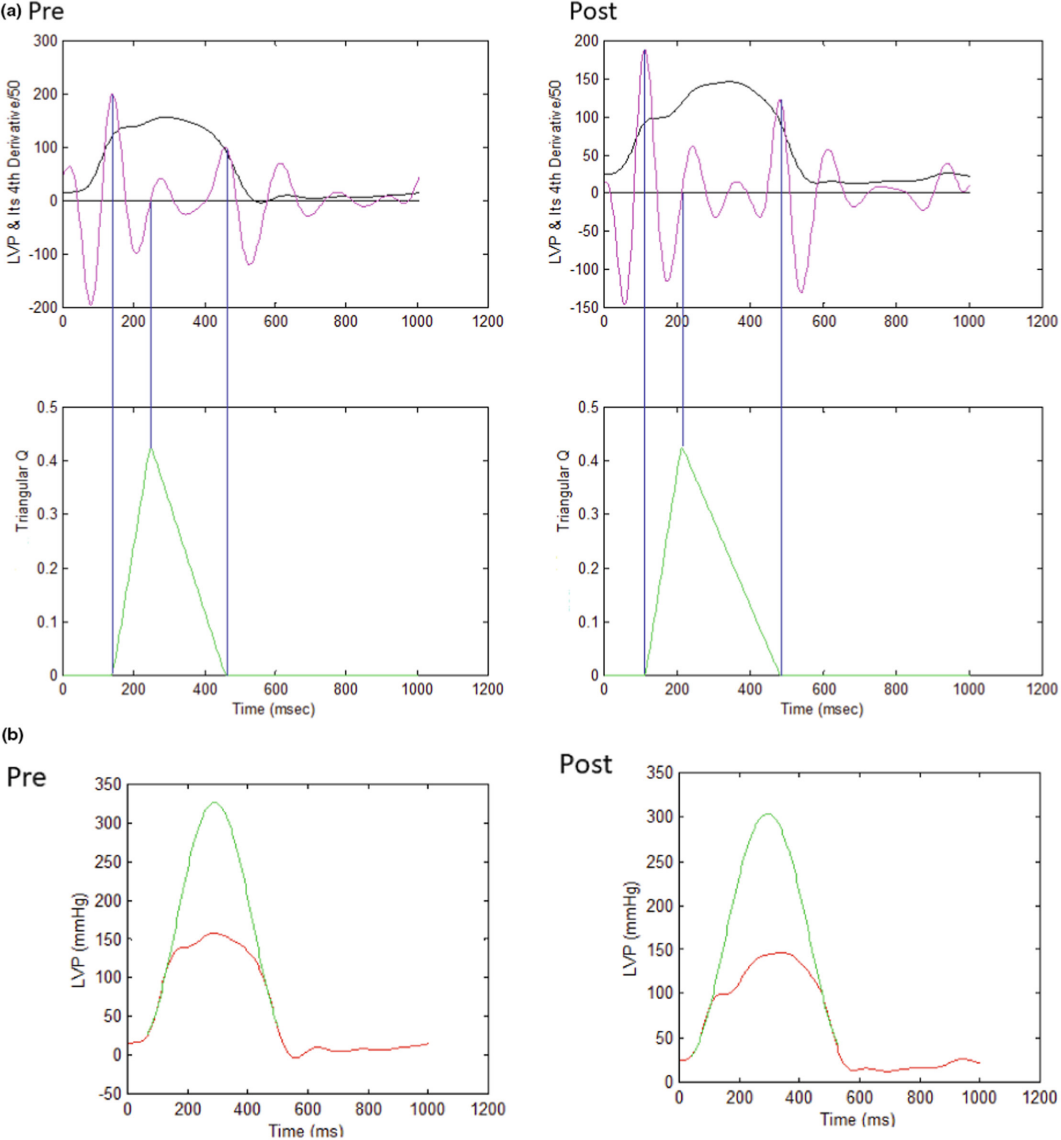
(a) Construction of Q^tri^ (green curve) from the fourth‐order derivative (pink curve) of the measured LVP (black curve). Q^tri^ onset was identified as the peak of the pink curve near the end of the isovolumic contraction period (first vertical blue line). Q^tri^ termination was identified as the nadir of the pink curve near the middle of the isovolumic relaxation period (third vertical blue line). The base of the unknown Q^tri^ was subsequently constructed with the duration being same as the time interval between the onset and termination of Q^tri^. After the ejection commenced, the first zero crossing from negative to positive (second vertical blue line) determined the peak of triangle. LVP, left ventricular pressure; Q^tri^, triangular blood flow. (b) Construction of LV isovolumic pressure (green curve) from measured LV pressure (red curve) with curve‐fitting method. LV, left ventricle.

**FIGURE 2:**
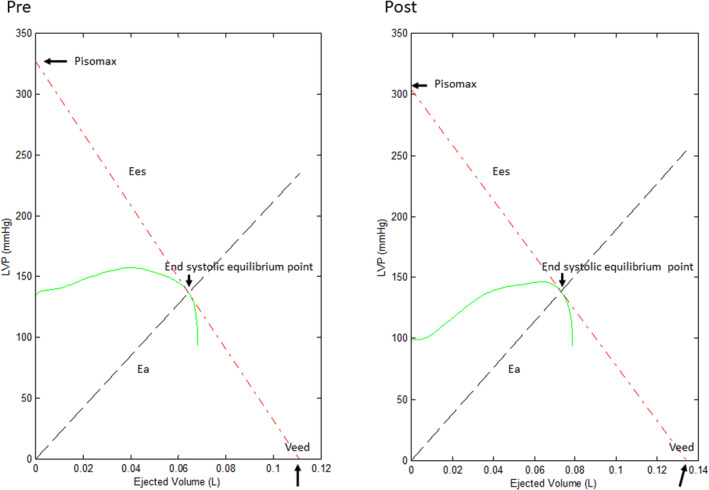
Construction of LV pressure‐ejected volume loop via Figure 1a,b. The Ees can be calculated as the slope of the tangential line which start from Pisomax to the right corner of pressure‐ejected volume loop which represents the end‐systolic equilibrium point. The Ea can be calculated from the slope of the original point to the end‐systolic equilibrium point. LV, left ventricle; Ees, end‐systolic elastance; Pisomax, LV peak isovolumic pressure; Ea, effective arterial elastance. Veed, effective LV end diastolic volume.

Traditionally, the PV analysis required multiple PV loops for calculation. The Ees is the slope of LV end‐systolic PV relationship. Theoretically, if an isovolumic LV PV curve could be constructed, this single PV loop could describe the properties of LV. We constructed the LV isovolumic contraction from an ejecting contraction to study the PV relationship by a method proposed in our previous studies (Wang et al., 2017, 2019). Two steps were needed for the construction. Firstly, we used the fourth‐order derivative of LV pressure to generate a triangular blood flow (Q^tri^) as an assumed flow of LV before and after TAVI (Figure 1a) (Kelly et al., 1989; Westerhof et al., 2006). The Q^tri^ was further adjusted according to the value of cardiac output. Secondly, we derived the estimated LV isovolumic pressure P_iso_(t) from a non‐linear least‐squares approximation technique with curve‐fitting method (Figure 1b) (Sunagawa et al., 1980). The constructed pressure‐ejected volume curve by the LV pressure and Q^tri^ was shown in Figure 2.

### 2.4 Calculation of the Ees and Ea from the constructed pressure‐ejected volume curve

By analyzing the constructed pressure‐ejected volume curve, the Ees is the slope from the point indicating estimated peak LV isovolumic pressure to the end systolic equilibrium point. The intercept of the Ees at the *x*‐axis was effective LV end‐diastolic volume (Veed). Veed is effective end‐diastolic volume = LV end‐diastolic volume—V_0_. The slope from the original point to the end‐systolic equilibrium point was Ea, the results are shown in Figure 2.

### 2.5 VAC and LV mechanics

Ea/Ees is widely used to represent VAC (Kelly et al., 1989). The stenotic valve and arterial system were integrated as an elastic chamber with specific elastance (Ea). As the ventricular elastance is connected to the arterial elastance, the energy transfer from LV to aorta had its coefficient. The total mechanical energy generated by LV can be presented by the pressure‐volume area (PVA). The efficiency of LV can be presented as the LV stroke work (SW) over PVA (Kass & Kelly, 1992).

The SW and PVA were derived from pressure volume loop and end‐systolic pressure volume relationship. In our constructed pressure‐ejected volume curve, the SW equals to the area under the measured LV pressure‐ejected volume curve. The PVA is the triangular area formed by the connecting the estimated peak isovolumic pressure and Veed points (Figure 2).

The maximal extraction of the external work from a given ventricle can also be used to represent the optimal energy transferal. The load condition (Q load) is determined from the SW to the theoretically maximal value (Kubota et al., 1992). The Q load can be calculated from Ea and Ees, with the equation below:

$$Q\;\text{load}=\frac{4\;\mathrm{Ea}/\mathrm{Ees}}{{\left(1+\mathrm{Ea}/\mathrm{Ees}\right)}^{2}}$$

The Q load is load‐independent, and derived from the function of Ea/Ees. The ideal value of Q load is 1.0 in theory, representing the condition of optimal energy transferal from LV to aorta.

### 2.6 Statistical analysis

The analysis was performed by STATA version 14.2 (StataCorp). The results were presented as mean ± standard deviation (SD) for continuous variables, and percentage for categorical variables. The comparison of pre and post TAVI data were performed by paired *t*‐test for continuous variables. The hypothesis of this pilot study was that the Ea would decrease by 0.8 following TAVI, and the estimated SD was 0.8. To achieve a power of 80% and level of significance 5%, the estimated sample size was determined to be at least 11. A *p* < 0.05 was considered statistically significant.

## 3 RESULTS

From September 2019 to August 2021, total 26 patients were included for analysis. The baseline and procedural characteristics are presented in Table 1. Mean age was 82.6 ± 7.9 years. 65.4% had hypertension, 42.3% had diabetes mellitus, 30.8% had chronic kidney disease, and coronary artery disease. The LV ejection fraction was 65.8 ± 12.6%. The peak and mean pressure gradients were 64.9 ± 13.4 and 38.0 ± 15.2 mmHg, respectively. The aortic valve area was 0.80 ± 0.17 cm^2^. The Zva was 2.4 ± 0.9 mmHg/mL.

**TABLE 1:** Baseline and procedural characteristics (*n* = 26).

Demographic data	
Age (years)	82.6 ± 7.9
Male	13 (50.0)
Stage of aortic stenosis	
D1	14 (53.8)
D2	4 (15.4)
D3	8 (30.8)
BMI, kg/m^2^	24.1 ± 4.3
STS score	3.2 ± 2.3
Hypertension	17 (65.4)
Diabetes mellitus	11 (42.3)
Coronary artery disease	8 (30.8)
Chronic kidney disease	8 (30.8)
Beta‐blocker	12 (46.2)
Calcium channel blocker	6 (23.1)
ACE‐I or ARB	12 (46.2)
LVEDV, mL	122.2 ± 41.5
LVEF, %	65.8 ± 12.6
AV peak PG, mmHg	64.9 ± 13.4
AV mean PG, mmHg	38.0 ± 15.2
Aortic valve area, cm^2^	0.80 ± 0.17
Zva, mmHg/mL	2.4 ± 0.9
Procedural characteristics	
Conscious sedation	25 (96.2)
Short‐acting vasopressor	24 (92.3)
THV type	
Evolut R	10 (38.5)
Sapien S3	16 (61.5)
THV size	
29 mm	6 (23.1)
26 mm	12 (46.2)
23 mm	8 (30.7)
Post‐TAVI AR	
None or trivial	20 (76.9)
Mild	6 (23.1)

*Note*: Values are mean ± SD or *n* (%).

Abbreviations: ACE‐I, angiotensin converting enzyme inhibitor; AR, aortic regurgitation; ARB, angiotensin II receptor blocker; AV peak PG, aortic valve peak pressure gradient; BMI, body mass index; LVEDV, left ventricular end diastolic volume; LVEF, left ventricular ejection fraction; STS, society of thoracic surgeon; THV, transcatheter heart valve; Zva, valvulo‐arterial impedance.

96.2% received consciousness sedation for TAVI. 61.5% of patients received Sapien S3 (Edwards Lifesciences), and 38.5% received Evolut R (Medtronic)

### 3.1 Acute change of hemodynamic parameters and results of PV analysis

The acute change of hemodynamic parameters after TAVI are summarized in Table 2. The heart rate and cardiac output increased after TAVI (heart rate, 69.3 ± 15.0 vs. 73.3 ± 14.8, *p* = 0.0197; cardiac output, 3.7 ± 0.8 vs. 4.3 ± 1.1 L/min, *p* = 0.0004). The LV end‐systolic pressure (LVPes) (162.7 ± 25.2 vs. 142.4 ± 33.2 mmHg, *p* = 0.0037) and LV peak systolic pressure (LVPmax) (173.8 ± 24.3 vs. 155.2 ± 31.1 mmHg, *p* = 0.0052) decreased after TAVI. The isovolumic peak LV pressure (LVPisomax) did not change after TAVI (274.9 ± 62.8 vs. 273.3 ± 67.3 mmHg, *p* = 0.8786). The stroke volume increased after TAVI (47.7 ± 12.1 vs. 54.0 ± 15.9 mL, *p* = 0.0057). Ea decreased after TAVI (3.7 ± 1.3 vs. 2.9 ± 1.1 mmHg/mL, *p* < 0.0001), while Ees did not change (2.4 ± 1.3 vs. 2.6 ± 1.1 mmHg/mL, *p* = 0.3670). The value of Ea/Ees and Q load both improved after TAVI (Ea/Ees: 1.8 ± 0.8 vs. 1.2 ± 0.4, *p* < 0.0001; Q_load: 0.89 ± 0.09 vs. 0.97 ± 0.03, *p* = 0.0001), demonstrating immediate improvement of VAC. The SW did not change (7669.6 ± 1913.8 vs. 7626.2 ± 2546.9 mmHg × mL, *p* = 0.9330), while PV area (PVA) decreased after TAVI (14469.0 ± 4974.1 vs. 12177.4 ± 4499.9 mmHg × mL, *p* = 0.0374), SW/PVA increased after TAVI (0.55 ± 0.12 vs. 0.63 ± 0.08, *p* < 0.0001), demonstrating the improvement of LV efficiency.

**TABLE 2:** Hemodynamic parameters Pre‐ and Post‐ TAVI.

*N* = 26	Pre‐TAVI	Post‐TAVI	*p* value
PCWP, mmHg	16.4 ± 9.9	18.2 ± 9.4	0.2688
PASP, mmHg	43.9 ± 17.8	42.3 ± 13.4	0.5962
RAP, mmHg	9.3 ± 5.9	10.6 ± 6.7	0.2881
Heart rate, beats/min	69.3 ± 15.0	73.3 ± 14.8	0.0197
Cardiac output, L/min	3.7 ± 0.8	4.3 ± 1.1	0.0004
Cardiac index, L/min/m^2^	2.3 ± 0.4	2.6 ± 0.6	0.0004
LVPes, mmHg	162.7 ± 25.2	142.4 ± 33.2	0.0037
LVPmax, mmHg	173.8 ± 24.3	155.2 ± 31.1	0.0052
LVPisomax, mmHg	274.9 ± 62.8	273.3 ± 67.3	0.8786
Stroke volume, mL	47.7 ± 12.1	54.0 ± 15.9	0.0057
Ea, mmHg/mL	3.7 ± 1.3	2.9 ± 1.1	<0.0001
Ees, mmHg/mL	2.4 ± 1.3	2.6 ± 1.1	0.3670
Ea/Ees	1.8 ± 0.8	1.2 ± 0.4	<0.0001
Q_load	0.89 ± 0.09	0.97 ± 0.03	0.0001
Veed, mL	129.2 ± 40.5	116.6 ± 34.5	0.0592
Stroke work, mmHg × mL	7669.6 ± 1913.8	7626.2 ± 2546.9	0.933
PVA, mmHg × mL	14469.0 ± 4974.1	12177.4 ± 4499.9	0.0374
Stroke work/PVA	0.55 ± 0.12	0.63 ± 0.08	<0.0001

*Note*: Values are mean ± SD or *n* (%).

Abbreviations: Ea, effective arterial elastance; Ees, LV end‐systolic elastance; LVPes, end‐systolic LV pressure; LVPisomax, peak isovolumic LV pressure; LVPmax, peak LV pressure; PASP, pulmonary arterial systolic pressure; PCWP, pulmonary capillary wedge pressure; PVA, pressure‐volume area; RAP, right atrial pressure; RVSP, right ventricular systolic pressure; Veed, effective LV end‐diastolic volume.

## 4 DISCUSSION

In patients with AS, left ventricle is contracting against the stenotic valve and the arterial load. Previous studies had discussed the load condition, but VAC was neglected (Briand et al., 2005; Ross Jr. & Peterson, 1973). We adopted a novel minimal invasive method to construct the PV loop in patients undergoing TAVI by analyzing LV pressure and an assumed flow. We demonstrated the improvement of Ea and VAC immediately after TAVI. The improvement of VAC was demonstrated in the change of Ea/Ees and the loading condition (Q load), both toward the ideal value of 1.0, representing better work transmission from LV to aorta. The value of SW/PVA also increased, representing the improvement of LV contractile efficiency after TAVI.

### 4.1 PV analysis in patients receiving TAVI

The comprehensive PV analysis can provide detailed information of pathophysiology. Currently the diagnosis and risk stratifications of AS are based on non‐invasive echocardiographic measurements and clinical symptoms. However, the decisions could be inappropriate in missing the timing of intervention in patients with marginal aortic valve area and pressure gradient, or at their later stage of AS due to LV dysfunction. Fundamental physiologic characteristics such as Ea, Ees, and VAC, may actually provide more detailed information for the pathophysiology of AS. Analysis of PV loop offered more in‐depth information of myocardial damage and contractility than echocardiography (Ishikawa et al., 2012). The VAC is a matching condition between ventricle and arterial system. Suga and Sagawa first adopted the PV relationship, they used Ees to describe the character of LV and Ea for the arterial system (Suga & Sagawa, 1974). Ea/Ees, or its function Q load were adopted to describe VAC (Kubota et al., 1992). In patients with AS, the LV contracted against the load from the stenotic valve and arterial system. TAVI relieved the valvular stenosis, resulted in significant improvement of afterload as seen in our study. The acute effect of afterload reduction was mainly attributable to the valvular component, thus the post‐TAVI Ea decreased significantly. The value of Ees did not change after TAVI. The improvement of VAC was mainly contributed to the decrease of Ea. The recovery of LV function and remodeling may occurre although not immediately but later within 1‐year (Kamperidis et al., 2014; Ribeiro et al., 2018). When the VAC improved, the work transferal from LV to arterial system moved toward ideal condition. The load condition could be presented as Q load, the optimal load condition defined as the condition allowing maximal energy transferal from LV to the aorta (Burkhoff & Sagawa, 1986; Kubota et al., 1992). When Ea/Ees equals to 1.0, the Q load reached its ideal value (ideal value = 1.0). As seen in our study, the Q load increased significantly after TAVI (0.89 ± 0.09–0.97 ± 0.03, *p* = 0.0001), demonstrating the improvement of energy transferal immediately after TAVI. Our study also showed the immediately improvement of LV efficiency (SW/PVA from 0.55 ± 0.12 to 0.63 ± 0.08, *p* < 0.0001). The SW equals to the LV systolic pressure × stroke volume. In our study, the LV systolic pressure decreased and stroke volume increased immediately after TAVI, thus the SW did not change significantly. There might also be partial effects of short‐acting vasopressor that could lead to incomplete resolution of LV pressure, thus the SW did not change significantly immediately after TAVI. Although the SW did not change, the improvement of LV efficiency was seen immediately. which was contributed to the decrease of PVA after TAVI. The improvement of energy transfer and LV efficiency were consistent with previous studies (Bastos et al., 2019; Marino et al., 2020).

Conventional method of PV analysis has major limitations such as the complexity for pressure/flow acquisition and its invasiveness. Our method is less invasive and simpler compared with conventional invasive method for PV analysis. However, our method developed from the mice study, using the disease model of diastolic heart failure. The accuracy of our model in AS required further study to validate. Our study is a proof of concept study. Immediately after TAVI, the Ea decreased while the Ees did not change. The post‐TAVI VAC and LV contractile efficiency improved immediately which is compatible with previous studies (Marino et al., 2020).

## 5 STUDY LIMITATIONS

The major limitation of the present study was the lack of good validation method. We did not perform conventional PV analysis due to its invasiveness and complexity. Also, our sample size was small and lack of long‐term follow‐up data. The clinical impacts and the long‐term results of the hemodynamic data were uncertain. Further study for clinical outcome comparison was warranted.

## 6 CONCLUSIONS

We demonstrated a minimally invasive method for PV analysis in patients with severe AS receiving TAVI. The Ea and VAC improved immediately after TAVI. The LV contractile efficiency improved which contributed to the decrease of PVA.

## AUTHOR CONTRIBUTIONS

Tsung‐Yu Ko contributed to planning/collecting data/writing. Chia‐Chuan Chuang/Kuo‐Chu Chang contributed to data analysis and collection. Mao‐Shin Lin/Yi‐Chang Chen/Ying‐Hsien Chen/Ching‐Chang Huang/Chih‐Fan Yeh/Ming‐Jiuh Wang contributed to collecting data. Hsien‐Li Kao/Yi‐Lwun Ho contributed to planning/writing.

## FUNDING INFORMATION

No funding information provided.

## CONFLICT OF INTEREST STATEMENT

The authors declare that the research was conducted in the absence of any commercial or financial relationships that could be construed as a potential conflict of interest.
